# Dengue Virus Capsid Protein Dynamics Reveals Spatially Heterogeneous Motion in Live-Infected-Cells

**DOI:** 10.1038/s41598-020-65625-6

**Published:** 2020-05-29

**Authors:** Manuela Gabriel, Guadalupe S. Costa Navarro, Luana de Borba, Andrés H. Rossi, Andrea V. Gamarnik, Laura C. Estrada

**Affiliations:** 1 Departamento de Física, Facultad de Ciencias Exactas y Naturales, Universidad de Buenos Aires and IFIBA-National Research Council for Science and Technology (CONICET), Buenos Aires, 1428 Argentina; 2 Fundación Instituto Leloir-National Research Council for Science and Technology (CONICET), Buenos Aires, 1405 Argentina

**Keywords:** Confocal microscopy, Biological fluorescence, Biological physics

## Abstract

Dengue is the single most important human viral infection transmitted by insects. The function of the viral proteins andtheir interactions with the host cell is under exhaustive investigation with the aim of identifying antiviral strategies. Here,using recombinant full-length dengue virus genomes, carrying a fluorescent mCherry fused to capsid, we studied biophysicalproperties of the viral protein during one infectious cycle in living cells. Dengue virus capsid protein associates to differentcellular compartments but its function in these locations is largely unknown. We evaluated the diffusion of capsid inside the celland determined a higher effective diffusion coefficient in the cytoplasm than in the nucleus. Using advanced fluorescencecorrelation methods, including the recently developed two-dimensional pair correlation analysis, we constructed for the first timehigh resolution maps of capsid mobility in an infected cell. We observed that the motion of capsid in the nucleoplasm-nucleolusinterface was highly organized, indicating an obstacle in this interface. Although nucleoli are membraneless structures, theydisplayed liquid-liquid phase separation. Once inside nucleoli, the protein showed isotropic mobility, indicating free diffusion orimmobilized capsid inside these structures. This is the first study presenting spatial and temporal dynamics of the dengue viruscapsid protein during infection.

## Introduction

Dengue virus (DENV) is the most significant arthropod-borne viral infection in humans. This virus belongs to the flavivirus genus, together with other important human pathogens, including Zika, Yellow Fever and West Nile viruses. Dengue is a mosquito-borne virus that causes large outbreaks and epidemics around the world. In the Americas, the number of dengue infections alarmingly increased in the last decade^1^. Despite the urgent need of controlling DENV infections, lack of understanding of molecular mechanisms of viral replication and interaction with the host cell limits the development of antiviral strategies. In this study, we investigated properties of one of the DENV structural proteins, the capsid (C) protein. This protein participates in two fundamental processes in the viral life cycle: genome encapsidation and genome uncoating. However, the mechanisms by which the C protein functions in these processes is largely unknown (for review see reference^2^).

**Figure 1:**
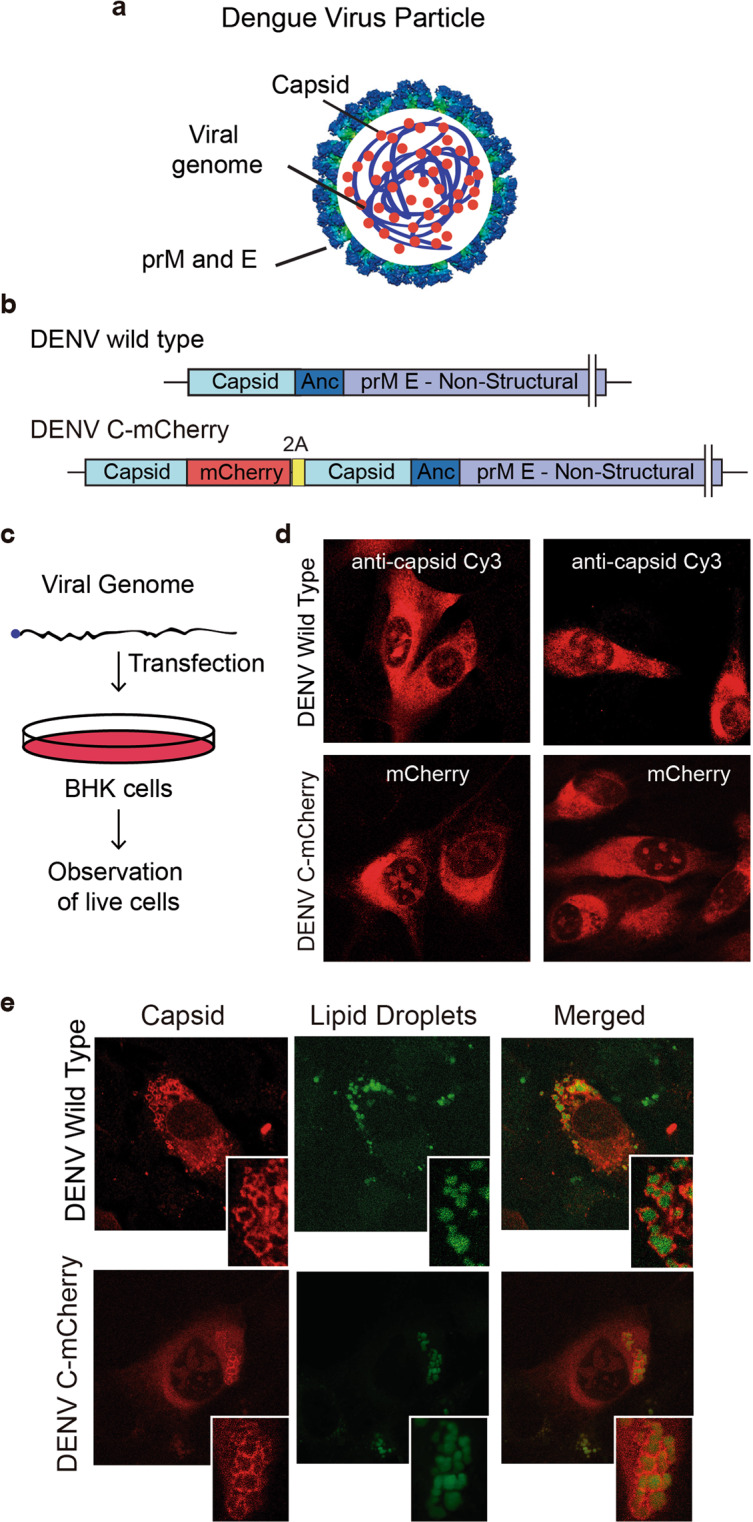
(**a**) Schematic representation of DENV particle showing the viral genome (blue), the capsid protein (red), and the lipid membrane containing the envelope (E) and pro-membrane (prM) proteins. (**b**) Representation of the DENV virus genome (DENV wild type) and the recombinant DENV carrying the capsid sequence fused to mCherry, followed by the food and mouth disease virus 2 A auto cleavage site. The complete sequence of the viral genome is indicated as prM E Non Structural region. (**c**) Protocol of viral RNA transfection. (**d**) Immunofluorescence using anti-C antibodies (DENV wild type) and C-mCherry visualization (DENV C-mCherry), both 24 h post-RNA transfection. (**e**) Immunofluorescence using anti-C antibodies (DENV wild type), C-mCherry visualization (DENV C-mCherry), and lipid droplets staining with BODIPY, both 24 h post-RNA transfection.

The DENV particle consists of a core formed by the nucleocapsid surrounded by a lipid bilayer containing viral structural proteins (Fig. 1a). The nucleocapsid contains the viral RNA genome, of positive polarity and single stranded, in complex with multiple copies of the C protein. The infection initiates by viral particle attachment to the host cell surface followed by receptor-mediated endocytosis. After entry, the viral membrane fuses with the endosomal membrane, releasing the viral genome into the cytoplasm for translation of viral proteins. The infection induces a profound rearrangement of cellular membranes that originates the so called replication complexes, in which genome amplification takes place by the viral RNA dependent RNA polymerase. Viral particle assembly occurs on the endoplasmic reticulum (ER) membrane, where the C protein recruits the viral genome to form the nucleocapsid, which in turn buds into the ER gaining the membrane containing the viral envelope and membrane proteins. The newly formed viral particles travel through the secretory pathway to be released outside the cell by exocytosis^3^. Although the primary function of the C protein in genome encapsidation takes place on the ER membranes, early after DENV infection the viral protein also accumulates in nucleoli and lipid droplets (LD), however, why and how capsid localizes in these compartments during infection remains unknown. In this context, fluorescence labeling of target viral proteins provides opportunities to visualize their intracellular distribution, dynamics and interactions.

In the last years, many fluorescence-based techniques have been developed to study molecular dynamics. Among others, fluorescence correlation methods are a family of microscopic approaches that provide information about the dynamics of fluorescently labeled molecules from the intensity fluctuations encoded in the emitted signal. Fluorescence intensity fluctuations in a single point (pFCS) inside a cell can arise from several different biological processes such as the diffusion of molecules through the observation volume, molecular conformational changes, binding-unbinding processes, or molecular transport, among others^4,5^. The fluorescence fluctuation autocorrelation function (ACF) calculated over the experiment time, is then fitted by analytical models of diffusion according to different biological processes^6,7^. As the observation volume is stationary in one position, pFCS provides dynamic information at a single location in the sample, of the size of the observation volume. In order to extent the study to the whole cell, the observation volume can be scanned relative to the sample and the ACF calculated from the intensity fluctuations at the pixels over an entire image^8–10^. Raster Image Correlation Spectroscopy (RICS) appears as a very convenient method to capture the subtle intensity fluctuations due to molecular dynamics and transport in an entire region or compartment of live cells. In this work, we used RICS to extract information about the average mobility of DENV C protein in cytoplasm and nucleus during the infection cycle.

Because, for most intracellular cases, molecules move in environments where obstacles to diffusion might exist, the possibility to visualize the path followed by molecules could unveil new aspects of their functions. Recently, a method based on cross correlating the intensity fluctuations at specific points in an image was proposed^11^. By correlating the signal at every pair of pixels, the two-dimensional pair Correlation Function (2D-pCF) determines the time for a particle to go from one location to another, allowing to create a visual map of the molecular average trajectories. This represents a breakthrough in the understanding of cell spatial organization and molecular functions.

Here, we applied the 2D-pCF analysis to create high resolution maps of C protein diffusion dynamics and transport, allowing for the first time the determination of the spatial communication inside the imaging area. Our results reveal a highly heterogeneous motion of DENV C protein inside live-infected-cells.

## Results

### Spatio-temporal localization of DENV capsid during infection

It has been previously reported that the DENV C protein associates to different cellular compartments during infection^12–15^. It has been shown that C accumulates in the nucleus and cytoplasm, and in the cytoplasm it distributes between the ER membranes and the surface of LDs^16^. In order to analyze the dynamics of C protein in vivo during one infectious viral cycle, we generated a recombinant full-length viral genomic construct encoding a C protein fused to mCherry coding sequence, in the context of a genome with a duplicated C coding region to ensure the function of RNA cis-acting elements (Fig. 1b)^17^. Transfections of transcribed viral RNA into mosquito or mammalian cells led to amplification of the viral genome and productive infections. In order to examine whether the fusion C-mCherry protein behaves as the WT C protein, we performed viral RNA transfections with the WT and the recombinant viral genomes (Fig. 1c). Immunofluorescence analysis using specific antibodies against C protein for the WT virus resembled that observed with the C-mCherry, analyzed by direct visualization of the fluorescent protein (Fig. 1d,e). The viral protein C and C-mCherry were found in the cytoplasm, nucleolus and lipid droplets. Therefore, this system was employed to investigate the properties of C protein in a viral replication cycle.

To get further insight on C protein localization during infection, transfected mammalian cells with DENV C-mCherry RNA were analyzed from 0 to 6 h. This time window corresponds to viral protein translation and RNA amplification.

**Figure 2:**
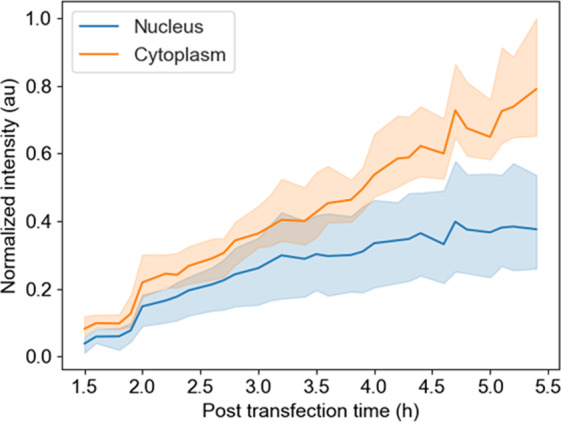
Normalized integrated fluorescence intensity in cytoplasm and nucleus of cells transfected with C-mCherry from 1.5 to 5.5 h time. Accumulation of C-mCherry in both compartments as a function of time is revealed. Mean fluorescence intensity (solid line) and standard deviation (shaded areas) in nucleus (blue) and cytoplasm (orange).

Real-time imaging during this time-lapse allowed analyzing the spatio-temporal localization of C-mCherry (Movie 1). The C protein was readily detected in the cell at 1,5 h after the onset of viral RNA translation. Interestingly, a rapid accumulation of C protein in the nucleolus was observed as early as 2 h after transfection. A distribution and accumulation of C-mCherry in the cytoplasm and nucleolus was observed as a function of time. A global picture of the protein intracellular spreading can be achieved if the integrated fluorescence intensity is calculated in time at different ROIs within the cell. The normalized average fluorescence time-dependence obtained at different locations in the cell (cytoplasm and nucleus) was measured (Fig. 2).

### Capsid protein dynamics in infected cells studied by raster image correlation spectroscopy (RICS)

To examine C protein dynamics, we characterized its average mobility in different intracellular compartments (cytoplasm and nucleus) during viral infection in BHK cells. For this, we used Raster Image Correlation Spectroscopy^10,18,19^ analysis of temporal fluorescence intensity fluctuations, which enabled us to obtain both high-time-resolution information on protein dynamics and spatial information of different mobility within the cell with subcellular (few *μ*m) spatial-resolution. In short, the RICS approach compares the intensity fluctuations of different pixels in an image of the region we want to study. Because the image is obtained by raster laser scanning, the time delay between one pixel and its neighbor in the horizontal and vertical directions are known (pixel dwell time and line time, respectively). If molecules that were in the first pixel move to the neighbor pixel in that amount of time, then there will be a positive correlation in the fluorescence fluctuations of these two pixels. This correlation will strongly depend on the mechanism driving the fluctuations of the measured signal. By comparing the fluctuations of different pixels in the image (this is, comparing points with different time delays), we can infer about the mechanism underlying the mobility of the molecules (see Methods section for further detail).

**Figure 3:**
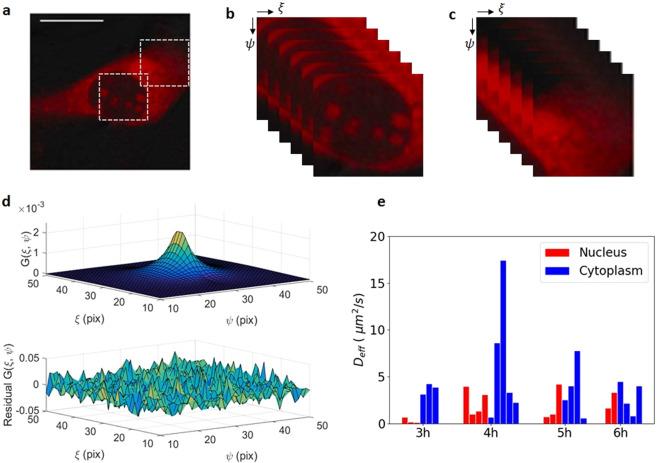
RICS measurement of C-mCherry diffusion. (**a**) Representative confocal image of a BHK cell transfected with C-mCherry (4 hours post transfection). A ROI of 256 × 256 pixels (12.8 × 12.8 *μm*^2^) is selected for RICS analysis in nucleus and cytoplasm. (**b**) Representation of image shifting for RICS analysis for the nucleus and (**c**) the cytoplasm. (**d**) Obtained fitted function of an image correlation result in a ROI after averaging 100 consecutive frames (top) and corresponding residual plot (bottom). Pixel shift starting at 0 is plotted from the center. (**e**) Effective diffusion coefficients obtained from the autocorrelation function of C-mCherry motion in nucleus and cytoplasm from 3 to 6 hours post transfection. Each bar represents a measurement in a single cell. 40 measurements from at least 5 independent cell cultures are shown.

Live-cell confocal images were acquired in BHK cells during DENV C-mCherry replication (Fig. 3). When a C-mCherry positive fluorescent cell was found, we zoomed on the cell, selecting different regions of interest for RICS measurements as indicated by the two squares in Fig. 3a. To improve the signal to noise ratio, for each region of interest, a stack of 100 consecutive images of 256 × 256 pixels (12.8 *μ*m × 12.8 *μ*m) was processed and the RICS ACF was obtained by averaging the spatial correlation functions of each of them. During a RICS experiment, excessive photobleaching could generate artifacts in the correlation curve. To avoid these artifacts laser power was set below 10%, and intensity traces checked before the analysis. Experiments with a bleaching >20% were excluded from this study. Because the sample is continuously imaged throughout the experiment, cell movement as well as the signal from fluorescent immobile objects were removed before performing the correlation (see Methods section). Finally, the 2D average spatial ACF (Fig. 3d, top) is calculated as a function of the pixel shift (which is the inter-pixel distance in the horizontal direction and the inter-line distance in the vertical direction).

Isotropic diffusion is the simplest model usually applied to proteins moving in the cellular environment. To compare the motion of C protein in different subcellular compartments, the ACF calculated from data obtained from cytoplasm and nucleus was fitted to a simple free diffusive mechanism. Figure 3d shows an example of a fitting function of the ACF (top) and the corresponding residual plot (bottom).

RICS experiments were repeated at different times during viral replication (3–6 hours). Prior to 2 hours, the fluorescence intensity was not high enough to obtain a good signal to noise ratio image. Since the determination of the parameters is based on curve fitting of the correlation function, the quality of the results strongly depends on how accurate the model describes the data. The goodness of the fitting curve is judged by the chi-square. In those cases when more than one set of parameters finds a minimum of the chi-square, measurements were excluded from further analysis.

After fitting, an average effective diffusion coefficient for each imaged area was obtained at different viral RNA transfection times (Fig. 3e). Each one of the vertical bars in Fig. 3e represents one successful fitting result, yielding values between 1–4 *μm*^2^/s in the nucleus, and 1–17 *μm*^2^/s in the cytoplasm. The mean values of the obtained effective diffusion coefficients Deff obtained at different hours post transfection (HPT) are summarized in Table 1. The ratio of both values indicates that, in average, C-mCherry moves “faster” in the cytoplasm than in the nucleus.

**Table 1:** Averaged effective diffusion coefficients (Deff) given in *μm*^2^/s obtained from the fitting of the correlation function by isotropic diffusion model in nucleus and cytoplasm at different hours post viral RNA transfection (HPT) are shown. The results are based on 40 successful fittings from 80 measurements.

HPT	Nucleus	Cytoplasm	Cyto/Nuc
3	1.0	3.8	3.8
4	2.3	6.5	2.8
5	2.0	3.7	1.9
6	2.5	2.9	1.2

The different diffusion coefficient for C-mCherry observed in the cytoplasm as a function of time is likely due to different capsid-protein interactions during viral replication, since the control mCherry diffusion coefficient did not change as a function of time. Nevertheless, we cannot rule out that cellular stress, such as endoplasmic reticulum stress, apoptosis, or other processes, alter viral protein behavior in the infected cell.

As control, RICS analysis in non-transfected cells showed minimal autofluorescence signal and no correlations were detected. Also, RICS experiments performed with mCherry expressing cells, revealed autocorrelation functions providing an effective diffusion coefficient between 1 and 20 *μm*^2^/s, which is consistent with the diffusion coefficient of similar fluorescent proteins reported previously^20,21^. Similar results were obtained in transfected cells between 3 to 5 hours post transfection time.

In order to visualize how the C protein interacts with the intracellular environment we applied the 2D-pCF method.

### DENV capsid protein intracellular dynamics and diffusion maps by 2D pair correlation function

The 2D pair Correlation Function approach creates a unique molecular flow map of the trajectory followed by C protein allowing to study intracellular communication in live cells. In the one-dimension version, the Pair Correlation Function method (pCF)^22–24^ measures the correlation between a pair of points that are located further apart (at a distance *δ*_*r*_) in the scanned pattern over the sample (line or circle). The temporal correlation function can be calculated at different time delays and a maximum of the correlation function will appear at a time delay proportional to the average time a molecule takes to move between the two points. The pCF over a line, for example, has been used to determine molecular transport among cell compartments^25,26^. In the recently presented 2D version of this technique^11^, the correlation function is calculated between two points (or pixels) at different frames from a temporal stack of images. At each point in the image, the pCF is calculated in every direction, this is, between points at different angles. From the correlation function calculated in every direction, an angular pCF distribution is obtained for each point, and the anisotropy of this distribution tells about the probability that the molecules moved in a specific direction in that point (see Methods section). Thus, the average path followed by the molecules at each point of the image is obtained. The main advantage of this technique relies on the possibility of mapping the flow followed by single diffusing molecules and its resolution is not limited by the size of the imaged area. The pCF is sensitive to the spatial heterogeneity of the cellular compartment, which makes it suitable to detect obstacles to diffusion and see how molecules move around them.

In the original work Malacrida and coworkers^11^ used a single plane illumination setup (light sheet) and a camera-based detection. In the present work, we have extended the implementation of the 2D-pCF approach to a commercially laser scanning microscope with a photomultiplier tube which is nowadays available in many biophysics labs. Although the temporal resolution is not as high as the one reached with a camera, the scanning is fast enough (32 ms/frame) to detect molecular displacement between the pair of points.

**Figure 4:**
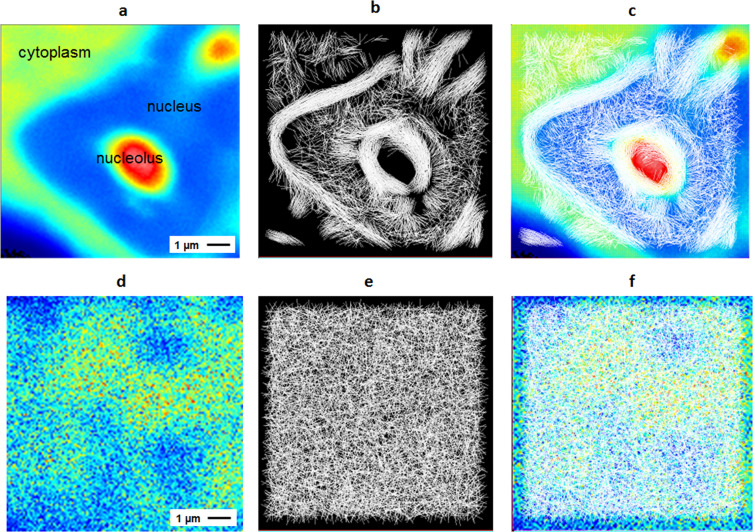
2D-pCF analysis calculated at 8 pixels (640 nm) distance and 6 hours post transfection. (**a**) Average intensity for the time series of a nucleus in a BHK cell transfected with C-mCherry. (**b**) Connectivity map represented with lines in the corresponding angle of higher anisotropy. (**c**) Average intensity of the series of frames superimposed with connectivity map. (**d**) Average intensity, (**e**) connectivity map and (**f**) connectivity map merged with intensity for the nucleus of a cell transfected with fluorescent protein mCherry.

A 2D-pCF analysis was calculated at a distance of 8 pixels (640 nm apart) applied to a stack of 8192 consecutive frames (Fig. 4). In all the experiments, images with a resolution of 128 × 128 pixels were taken. The average intensity from the complete stack of images from a nucleus was measured 6 hours post viral RNA transfection (Fig. 4a). Intensity scales is color-coded from red (highest) to blue (lowest). At this time scale, when viral translation and RNA replication are actively ongoing, the C protein heavily accumulates in nucleoli (bright red, Fig. 4a). In contrast, the nucleoplasm is visualized as a region of low intensity. In addition, the cytoplasm clearly shows higher concentration of that observed in the nucleoplasm, delineating an interface at the nuclear membrane.

To analyze the molecular flow of the C-mCherry, a line where the anisotropy direction is maximized was drawn in every pixel of the image (Fig. 4b). The map of the anisotropy direction describes the most probable paths the molecules follow at each pixel, indicating the molecular flow. This representation, also named the connectivity map, because it is sensitive to barriers and obstacles for diffusion, allows the visualization of a map representing the protein intracellular flow. The connectivity map was plotted over the average intensity of a series of frames (Fig. 4c). This merge lets us identify the diffusion direction in every region of the nucleus, making it evident that in the cytoplasm-nucleus and nucleoplasm-nucleolus interfaces, the molecule’s motion has a unique behavior.

As a control experiment, the same procedure was performed in cells expressing fluorescent mCherry protein alone. In this case, the protein is not expected to accumulate in a specific location, which is evident from the intensity image (Fig. 4d). The distribution of the intensity along the image shows the heterogeneity of the nuclear environment. However, the connectivity map obtained for this sample (Fig. 4e) shows a highly isotropic motion despite the heterogeneity in the chromatin distribution and the presence of nucleoli. This result also indicates that the changes in the intensity from one pixel to the one next to it is not generating artifacts in the connectivity map. Similar results were observed for all analyzed cells expressing mCherry. By comparison of Fig. 4b,e, it is evident that in DENV replicating cells expressing C-mCherry, there are regions of “ordered diffusion”, meaning that the anisotropy direction changes softly from pixel to pixel, while there are some regions where the distribution appears to be random (no visible organization in the molecular flow). From the merged images (Fig. 4c,f), we conclude that the ordered mobility occurs in specific regions of the cell and only when expressing C-mCherry, indicating specific behavior of the viral C protein in the cytoplasm-nucleus and nucleoplasm-nucleolus interfaces.

**Figure 5:**
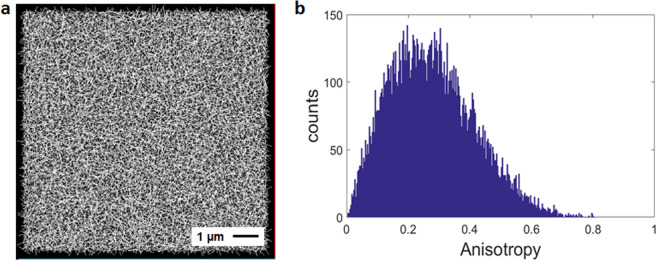
Anisotropy threshold. (**a**) Connectivity map and (**b**) anisotropy values histogram obtained for a solution of EGFP. Anisotropy mean value is 0.27 ± 0.13.

To build the diffusion maps shown in Fig. 4 the first step was to determine the anisotropy-threshold-value expected for random isotropic diffusion. To this end, we performed confocal raster-scanning measurements using a 10 nM solution of monomeric EGFP. 8192 images were taken and used to built the corresponding histogram of the anisotropy values. We applied the 2D-pCF analysis at each pixel of the image time series using 4 pixels distance (320 nm) and obtained the connectivity map and anisotropy histogram shown in Fig. 5a,b. A mean value of 0.27 ± 0.13 was obtained for random motion in the absence of obstacles to diffusion. This is considered in our analysis as a reference value to identify regions of random motion during data visualization, meaning that the anisotropy values below this threshold will be kept out of the analysis for molecular flow visualization.

**Figure 6:**
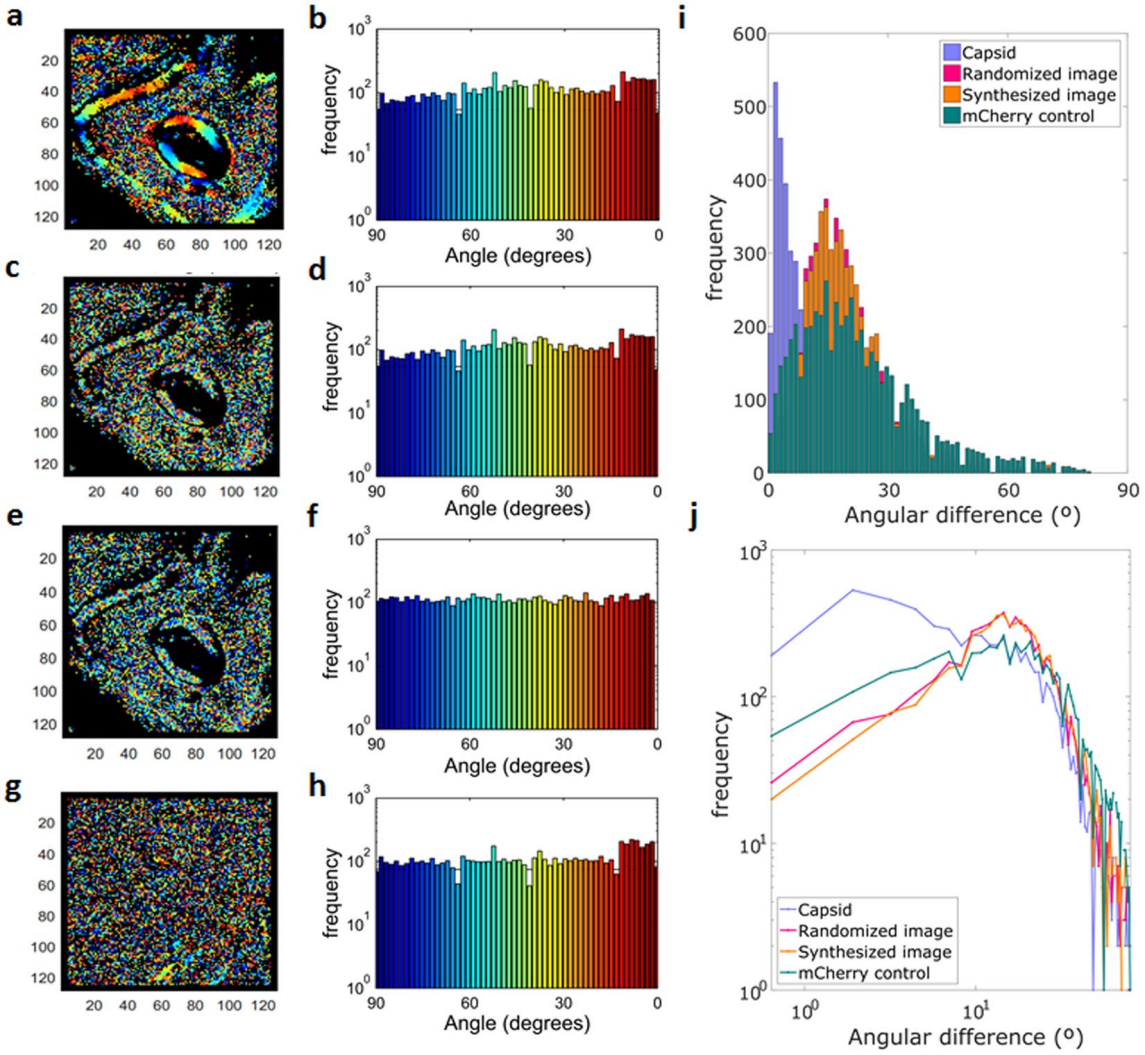
Anisotropy analysis. Color-coded representation of the direction of maximum anisotropy and their corresponding color distribution histograms for: (**a**,**b**) A ROI containing a nucleolus in a C-mCherry infected cell, (**c**,**d**) randomized image of colors in (**a**), (**e**,**f**) Synthetic image of random colors in the same range and (**g**,**h**) mCherry transfected cell. As mCherry do not specifically interact with nucleoli, these structures are not visualized as clear as in (**a**). (**i**) Distributions of the sum of the quadratic color differences between neighbor pixels and (**j**) same histogram in logarithmic scale.

The analysis presented in Fig. 4 indicates that, while mCherry in the nucleus diffuses isotropically, C-mCherry presents a dramatically different dynamic behavior. In order to quantitatively distinguish between these different outcomes, we studied the anisotropy distribution in the images. With this purpose, the anisotropy direction was indicated in a color-coded scale according to the angle of orientation (Fig. 6a). Each color represents the preferential direction of diffusion obtained from the anisotropy at each pixel, from red (horizontal or 0°) to blue (vertical or 90°). Then, the histogram for the color distribution, or anisotropy direction, was built (Fig. 6b). The quasi-uniform distribution shown in Fig. 6b reveals it is not possible to determine the prevalence of certain orientation. The required analysis for quantification of this effect, then, needs to contain spatial information. To this end, we created two additional images to compare with the real data. The first image (Fig. 6c) was constructed by randomly re-distributing the colors of the original anisotropy image, and the second one (Fig. 6e) is a synthetic image obtained by randomly assigning to each pixel a color, assuming a uniform distribution. Histograms of color distribution are shown. As it was expected, Fig. 6b,d show exactly the same distribution as they contain identical data. However, there are not significant differences with the histogram obtained when creating a random color distribution in the same range (Fig. 6f). As a biological-control experiment, the anisotropy map obtained from a cell expressing free mCherry, which was expected to undergo isotropic diffusion, was used (Fig. 6g,h).

The four histograms are not distinguishable, indicating that further analysis is required. The four images (C-mCherry, the randomized, the synthetic, and mCherry control) were compared by means of the quadratic color difference of consecutive pixels. This analysis consisted of calculating, for each non-black pixel in the image, the color difference (angular direction difference) between neighbor pixels in every direction. The resulting distributions of the sum of the quadratic color differences between neighbor pixels for the four analyzed images is shown in Fig. 6i. While the distributions for the two synthetic images with the control experiment are not statistically different, there is a significant difference (P < 0.001, two-tailed paired t-test) between them and the anisotropy distribution for the C-mCherry containing cells. The difference becomes clearer in a logarithmic scale plot (Fig. 6j). These results indicate that during DENV infection, there is a high contribution of small differences between colors of neighbor pixels, which does not occur when the colors are randomized in the image or when a new set of random colors are assigned, nor even in the control experiment with free mCherry.

**Figure 7:**
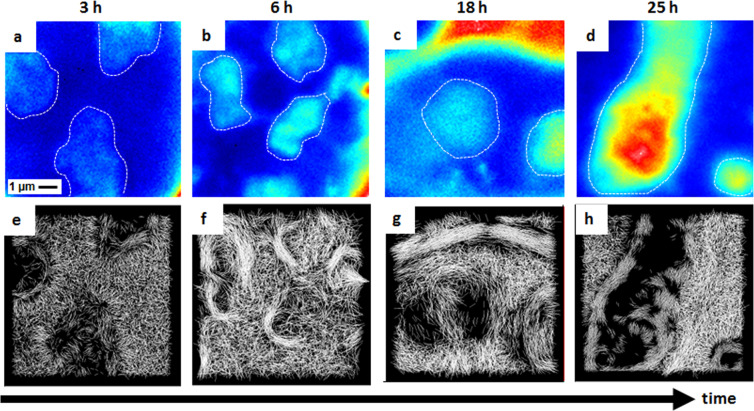
C-mCherry mobility in nucleus as a function of post-transfection time. (**a–d**) Average intensity nuclear image of four different cells at 3, 6, 18, and 25 hours post transfection, where nucleoli are visualized as regions of higher intensity (marked in dotted lines). (**e–h**) Corresponding connectivity maps calculated at a distance of 8 pixels (640 nm).

To obtain information about C protein dynamics during the course of infection, it was necessary to study its behavior as a function of time. We compared the diffusion connectivity maps obtained from the 2D-pCF analysis in the nucleus of different cells at 3, 6, 18 and 25 hours post genomic RNA transfection. Figure 7a–d show average intensity images, and Fig. 7e–h show the corresponding connectivity maps. Nucleoli can be identified as brighter regions inside the nucleus and are delineated with dashed white lines as reference. At 3 h post viral RNA transfection C-mCherry is diffusing in the nucleus and a few pixels of positive correlation can be identified inside nucleoli. At 6 h the movement of C-mCherry around nucleolus becomes evident, and at 18 and 25 h a circulation around nucleoli can be clearly observed.

To avoid that many lines overcrowd the connectivity map, we built maps using only the pixels with anisotropy values above the threshold and thus, the lack of connectivity observed in any pixel, indicates that the values found in that region are below the threshold. This could occur for various reasons: because the molecules are immobile (zero correlation), because they permanently bind to intracellular compartments, or because their motion is described by isotropic diffusion, among others. In our case of study, it is reasonable to expect that as C protein accumulates in nucleoli, its motion will be more restricted. This lack of movement can be due to permanent binding of the C protein with nucleolar components such as RNA and/or proteins, or due to the compact structure of nucleoli.

**Figure 8:**
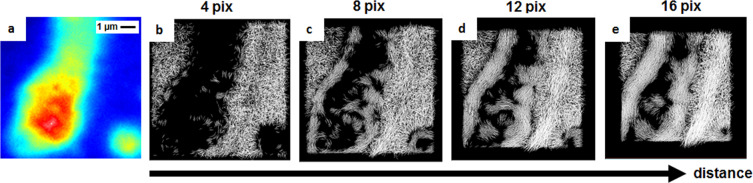
pCF calculated at increasing distances shows different obstacles to diffusion inside the nucleus. (**a**) Average intensity of imaged area (**b–e**) Connectivity maps calculated at a distance of 4, 8, 12 and 16 pixels.

The pCF analysis can be performed between any pair of pixels at any distance. The distance used to compute the correlation function determines the features the method will highlight. As an example, by changing the pixel distance, we will detect the correlation produced by molecules moving at different velocities^11^. For this reason, we calculated the 2D-pCF of the image in Fig. 8a for increasing pixel distances of 4, 8, 12 and 16 pixels (Fig. 8b–e) corresponding to 320 nm, 640 nm, 960 nm, and 1.28 *μm* respectively.

The difference in the connectivity maps calculated using the same stack of images at different pixel distances is evident. At 4 pixels distance, the connectivity map obtained from the pCF analysis shows an empty nucleoli interior and no organized movement around it. These results have two possible interpretations: (i) C-mCherry inside nucleoli is diffusing in a highly isotropic manner, or (ii) C-mCherry is not moving when inside nucleoli. The comparison between pCF(4) and pCF(16) (Fig. 8b,e) shows regions in the nucleoli where the anisotropy at larger distances becomes evident, suggesting the occurrence of barriers at the nucleoli-nucleoplasm interface, which are not visible in the short scale analysis with short pixel distances.

These studies revealed the molecular flow and the barriers that the C protein encounters in different cellular compartments during a viral infectious cycle.

## Discussion

The DENV C protein plays multiple functions and associates to several viral and host components in different compartments of the infected cell. However, the relevance of these interactions and the implications of its subcellular localization remain unknown. In this work, we revealed, for the first time, a spatially heterogenous mobility of DENV C protein in different cellular compartments during the infectious cycle. We applied advanced image correlation techniques that are non-invasive, do not need to perturb the system and achieve single molecule resolution in bulk experiments.

First, we used RICS to estimate the average spatial mobility of C protein in the cytoplasm and the nucleus. These results proved that C diffusion in each compartment have a different macroscopic diffusion coefficient, suggesting that C diffuses faster in cytoplasm than in nucleus during the first 6 hours of viral infection.

Second, to get further detail of the molecular mobility inside each region without the spatial average, we applied the 2D pair Correlation Function method. Although the pair Correlation Function is becoming a widely used method in the family of fluorescence correlation spectroscopy techniques, the two-dimensional approach has been introduced recently. From the correlation in every direction the diffusion anisotropy at every point of the image is obtained, meaning that the spatial resolution of this method is at the pixel level. From the 128 × 128 calculated anisotropy values, we created connectivity maps excluding those values below a certain threshold that were associated to isotropic diffusion. The connectivity maps allowed us to visualize the average path followed by C protein, showing a particular behavior in the proximity to the nuclear membrane and nucleoli, which was not seen with the non-interacting fluorescent protein.

The C protein was found accessing the nucleolus as early as 2 hours of the onset of viral translation, indicating that C molecules, translated from the incoming RNA, distribute between the cytoplasm and the nucleus, accumulating in nucleolus. The ordered motion of C protein observed at the cytoplasm-nucleus interface reflects the presence of the nuclear membrane as a physical barrier. When the protein enters the nucleus, it quickly associates to nucleolus. The molecular flow at the interface nucleoplasm-nucleolus indicates that the C protein also encounters an obstacle for diffusion, which forced the molecule to move around this structure. Despite this, the viral protein entered inside nucleoli and accumulated at high concentrations. Nucleoli are membraneless structures but organized by liquid-liquid phase separation. Although they are dynamic organelles, they have a densely packed structure. In this regard, the mobility of the C protein abruptly changes when it is found inside nucleolus. It is possible that C protein mobility is restricted by interactions with specific nucleolar components such as nucleic acids or proteins. Because the function of DENV C in nucleolus and other subcellular compartments is still unclear, and the dynamics of other viral proteins have not been examined, we propose to further explore the techniques presented here to understand biophysical and biological properties of viral proteins.

In summary, this is the first study that presents a high spatial and temporal resolution analysis of DENV C distribution and movement during viral infection in live cells using advanced correlation techniques on confocal images.

## Methods

### Construction of recombinant DENV C-mCherry

For constructing the recombinant full-length DENV containing the C-mCherry fusion, we modified a DENV reporter construct that we have previously described that includes a luciferase coding region^27^. We replaced the SacII-SphI fragment of the reporter with a fragment containing mCherry coding sequence fused to the food and mouth disease virus 2A (FMDV2A) protein, in such a way to obtain the capsid fused to mCherry that would be released from the rest of the viral polyprotein by the FMDV2A cleavage. The fragment used to replace the SacII-SphI region was obtained by overlapping PCR. The new construct named DENV C-mCherry was sequenced and directly used for *in vitro* RNA transcription. The viral RNA DENV C-mCherry was competent for viral translation and genome replication.

### Cell culture and virus transfection

Baby hamster kidney cell line (BHK-21) was cultured in minimum essential medium alpha (α-MEM) supplemented with 10% fetal bovine serum (Gibco), 100 U/ml penicillin-streptomycin. Plasmids containing DENV WT and DENV C-mCherry full genomes were linearized with XbaI restriction enzyme. Viral capped RNA was in vitro transcribed by T7 RNA polymerase in the presence of m7GpppA. RNA transfections were performed using Lipofectamine 2000 (Thermo Fisher Scientific) and Opti-MEM medium (Gibco) according to the manufacturer’s instructions. Fifty ng of viral RNA WT or C-mCherry were transfected into BHK-21 cells grown in 8-well chambered cover-glass plates (Thermo Fisher Scientific). The observations were performed immediately after adding transfection reagent. For the mCherry control experiments, 500 ng of pmCherry-C1 plasmid (Clontech) were transfected into BHK cells using the protocol described above

### Confocal microscopy of transfected BHK cells

For laser scanning confocal microscopy experiments, images were acquired with a LSM 880 (Zeiss) inverted microscope and a C-Apochromat 40X/1.2 water-immersion objective (Zeiss). All experiments were carried out using a detection pinhole size of 1 Airy disk unit. During the experiment time, cells were kept at 37 °C in a 5% CO_2_ temperature-controlled chamber (Pecon). Excitation wavelength was set at 543 nm using a He-Ne green laser (Lasos) and intensities chosen to achieve high photon count rate per molecule to maximize the signal/noise ratio but low enough to prevent molecular photobleaching and phototoxicity processes. The obtained photon count rates per molecule ranged from 1 kcps to 20 kcps approximately. Fluorescence emission was collected between 550 − 650 nm using a spectral filter (Quasar detector) and detection performed by GaAsp detector (Hamamatsu) in photon counting mode. For RICS measurements a sequential image series at a fixed pixel size of 50 nm and with a pixel dwell time of 16 *μ*s was used. Typically, images had 256 × 256 pixels (corresponding to an area of 12.8 × 12.8 *μ*m^2^) and a total of 100 frames at 1.62 s frame rate were collected for each measurement. For 2D-pCF analysis pixel time was 0.98 *μ*s, pixel size was 80 nm, images had 128 × 128 pixels (corresponding to an area of 10.2 × 10.2 *μm*^2^) and a total of 8192 frames at 0.35 s frame rate were taken for each measurement. In addition, the laser power was kept low enough to avoid bleaching higher than 10% so no detrend of the intensity was necessary. Data was analyzed using commercial software SimFCS (LFD, UCI, www.lfd.uci.edu) and custom-made code written in Matlab (MathWorks) and Python.

### Data analysis

#### Raster image correlation spectroscopy

In a typical RICS experiment, data is collected using a confocal (or two photon) laser scanning microscope. A sample is raster scanned with a pixel dwell time which is generally on the order of few microseconds and the pixel size is set to be smaller (about a factor of 3–5) than the waist of the PSF (Point Spread Function). Each line of the scan has duration in the millisecond range and the entire frame is acquired in times on the order of 1 s. One image is enough to calculate the RICS correlation function. However, to increase the statistic and, subtract the immobile features or very slow-motion objects in the image (for example due to the cell movement), a stack of about 50 to 100 images is generally acquired. The subtraction made in this analysis was considering a moving average, this is, the average of a certain range of consecutive frames is calculated and subtracted for the frame in the middle of this range. Every frame will have a subtraction except for a group of frames in the beginning and in the end of the time series. For example, if 100 frames are image and the moving average is calculated with 10 frames, then average between frames 1–10 will be subtracted from frame 5, doing so until frame 95 which will be the last one in the analysis.

After background subtraction, the 2D correlation operation is applied to each of the images of the stack. The average 2D spatial correlation is calculated using Eq. (1):

1 $${G}_{RICS}(\xi ,\varPsi )=\frac{\langle I(x,y)I(x+\xi ,y+\varPsi )\rangle }{\langle I(x,y)\rangle \langle I(x,y)\rangle }-1$$

Where *I*(*x*, *y*) is the intensity at pixel x, y of the image, *ξ* and *ψ* are the image shift for x and y directions respectively, and *δ*_*i*_ is the intensity fluctuation.

The correlation function results from the spatial average, and the RICS data is obtained after all analyzed frames are averaged. This data is finally fitted according to the equations that describe the molecules motion. This equation considers two factors: one given by scanning optics and the other one related to molecules motion. The most generalized model then is Eq. (2):

2 $${G}_{RICS}(\xi ,\varPsi )=S(\xi ,\varPsi )G(\xi ,\varPsi )+b$$

Where *b* is a background correction. For isotropic diffusion of molecules, the RICS function is fitted by the model presented in Eq. (3):

3 $$G(\xi ,\varPsi )=\frac{\gamma }{N}\left(1+{\frac{4D({\tau }_{P}\xi +{\tau }_{L}\varPsi )}{{\omega }_{0}^{2}}}^{-1}\right){\left(1+\frac{4D({\tau }_{P}\xi +{\tau }_{L}\varPsi )}{{\omega }_{z}^{2}}\right)}^{-1/2}$$

In this equation, *D* is the diffusion coefficient, *τ*_*p*_ and *τ*_*l*_ are the pixel dwell time and the line time respectively, *ω*_0_ and *ω*_*z*_ are the waist (1/e^2^) of the PSF in the radial and axial directions, *γ* is a factor that account for the profile of illumination (0.35 for 3D Gaussian and 0.076 for Gaussian Lorentzian) and *N* is the number of molecules in the excitation volume. If the pixel size is *δ*_*r*_, the scanning term is given by:

4 $$S(\xi ,\varPsi )=\text{exp}\left[\frac{{\left(\frac{\xi \delta r}{{\omega }_{0}}\right)}^{2}({\xi }^{2}+{\varPsi }^{2})}{1+\frac{4D({\tau }_{P}\xi +{\tau }_{L}\varPsi )}{{\omega }_{z}^{2}}}\right]$$

It is important to note that all the temporal information of RICS is within one frame. The analysis considers multiple images for statistical purposes. On one hand, the background subtraction needs to more than one frame to be done, and on the other hand the signal to noise ratio improves when the RICS function is obtained from the average of several frames.

#### pair correlation function

The 2D-pair Correlation Function (2D-pCF) approach is intended to determine the average path for a molecule diffusing in the cell interior with statistical spatio-temporal significance. Analog to the line pCF, where the correlation function is computed between points at different lines^28^, the 2D-pCF is computed between points from different images^11^. This is opposite to RICS, where one image is enough to compute the correlation function.

Closely analogous to the 1D case, the 2D-pCF at a given distance (*δ*_*r*_) is defined as:

5 $$pCF(\delta r,\tau )=\frac{\langle I(r,t)I(r+\delta r,t+\tau )\rangle }{\langle I(r,t)I(r+\delta r,t)\rangle }-1$$

Where *τ* is the acquisition time between the point *r*_0_ in one image and *r*_1_ in the other image separated by a distance of *δ*_*r*_, I is the recorded fluorescence intensity in each point of the image, and the brackets indicate a temporal average.

The 2D-pCF approach detects obstacles to diffusion by computing the delay time on the pair correlation-function-maximum amplitude (this is the case if there are interactions between the molecules and intracellular compartments) or, by the absence of a correlation between the two points (this will occur in the case of impenetrable barriers to diffusion). The pixel distance *δ*_*r*_ can be varied in order to visualize the path of molecules that travel different distances.

To calculate the 2D-pCF, a stack of at least 8192 images of the same focal plane is needed. From the stack of images, the pCF(*δ*_*r*_) at many angles around a given point for all the pixels in the entire image are calculated to determine the average molecular path to diffusion. The number of angles is given by 8 times *δ*_*r*_, meaning that for a distance of 4 pixels, the pCF is calculated over 32 angles around each point. This procedure defines a diffusion tensor for each pixel in the image and the pCF distribution is plotted in a polar plot. The moments of the intensity distribution *I*(*x*, *y*) can be calculated as follows:

6 $${M}_{i,j}=\frac{{\sum }_{x}{\sum }_{y}{x}^{i}{y}^{j}(x,y)}{{\sum }_{x}{\sum }_{y}I(x,y)}$$

Then, the first and second central moments (*μ*_11_, *μ*_20_ and *μ*_02_) of the spatial distribution can be calculated from the general equation for central moments:

7 $${\mu }_{p,q}=\sum _{x}\sum _{y}{(x-\bar{x})}^{p}{(y-\bar{y})}^{q}I(x,y)$$

Where $\bar{x}$ and $\bar{y}$ are the coordinates of the center of mass of the distribution. Then, the center of mass shift, the length of the distribution long and short axis and the angle with respect to the image coordinates can be calculated. The angle will indicate the preferably direction of molecular diffusion when the diffusion is anisotropic, and it is given by:

8 $$\theta =\frac{1}{2}\text{arctan}\left(\frac{{2}_{\mu 11}}{{\mu }_{20}-{\mu }_{02}}\right)$$

The length of the long (λ_1_) and short (λ_2_) axis of the distribution are defined as:

9 $${\lambda }_{1,2}=\frac{{\mu }_{20}+{\mu }_{02}}{2}\pm \frac{\sqrt{4{\mu }_{11}^{2}+{({\mu }_{20}-{\mu }_{02})}^{2}}}{2}$$

With these two parameters, the Anisotropy, which indicates the how asymmetrical is the pCF in each pixel, is calculated as:

10 $$A=\frac{{\lambda }_{1}-{\lambda }_{2}}{{\lambda }_{1}+{\lambda }_{2}}$$

Higher Anisotropy values indicate that the motion is less isotropic in the direction given by the angle calculated before. This analysis provides an angle and anisotropy value for each point of the image, which produces a map of the molecular flow in the whole illuminated region.

## Supplementary information

Supplementary information.
